# Immunomodulatory Mechanisms and Therapeutic Potential of Vitamin D in Immune Thrombocytopenia

**DOI:** 10.1155/jimr/5810208

**Published:** 2025-12-06

**Authors:** Yucao Ma, Wenjing Yao, Haiyan Lang, Yuxin Cheng, Ruhua Ren, Yuecan Chen, Sitong Cheng, Shuo Sun, Qing Guo, Shana Chen

**Affiliations:** ^1^ Department of Hematology, Dongzhimen Hospital, Beijing University of Chinese Medicine, Beijing, 100700, China, bucm.edu.cn; ^2^ Department of Hematology, International Mongolian Hospital of Inner Mongolia, Inner Mongolia Autonomous Region, Hohhot, 010020, China

**Keywords:** immunomodulation, ITP, review, vitamin D, vitamin D receptor

## Abstract

In addition to its classical function in regulating phosphorus and calcium equilibrium, the immunoregulatory effects of vitamin D (VD) have increasingly drawn attention in recent investigations. A deficiency of VD has been associated with multiple autoimmune pathologies. Immune thrombocytopenia (ITP), a hemorrhagic disorder mediated by autoantibodies, manifests through accelerated platelet destruction accompanied by impaired platelet production. Accumulating evidence suggests that VD is intricately involved in ITP pathophysiology, with serum VD concentrations strongly associated with clinical symptoms, disease severity, and overall prognosis. However, the mechanisms underlying these associations remain incompletely understood, and the limited number of studies conducted thus far highlights the necessity for further investigation. This study reviews the correlation between VD and ITP, the immunological mechanisms through which VD regulates ITP, and the potential therapeutic value of VD supplementation in ITP management. Future multicenter clinical trials and mechanistic studies are warranted to develop novel therapeutic strategies for ITP.

## 1. Introduction

Immune thrombocytopenia (ITP) is an autoimmune disorder characterized by diminished platelet counts and an increased risk of bleeding. Its etiology involves aberrant activation of both cellular and humoral immune mechanisms [1]. Present clinical approaches for managing ITP include glucocorticoids, intravenous immunoglobulin (IVIG), thrombopoietin receptor agonists (TPO‐RAs), immunosuppressive medications, targeted biological agents, and surgical interventions such as splenectomy. Despite these strategies, certain patients continue to experience therapeutic resistance or relapse, and the prolonged administration of such treatments may induce complications, including infections and metabolic disturbances [2, 3].

Numerous studies indicate that vitamin D (VD) functions beyond calcium‐phosphate metabolism, influencing the differentiation, proliferation, and function of various immune cell subsets via its active form, 1,25(OH)_2_D_3_, through interactions with VD receptor (VDR). VD supplementation has shown promising outcomes in preclinical and clinical studies addressing several autoimmune disorders, such as inflammatory bowel disease (IBD) and type 1 diabetes mellitus [4, 5]. Considering that VD insufficiency is frequently observed in ITP patients and correlates significantly with disease severity, clinical outcomes, and prognosis, further investigation into the therapeutic potential of VD in ITP is warranted. This study systematically reviews the immunomodulatory mechanisms and potential clinical benefits of VD in the context of ITP.

## 2. Overview of VD

VD synthesis primarily occurs in the skin, where ultraviolet B (UVB) radiation converts 7‐dehydrocholesterol (7‐DHC) into VD_3_ (cholecalciferol), accounting for more than 80% of the body’s total VD. Additionally, dietary sources such as fatty fish, egg yolks, and fortified foods supply VD_2_ (ergocalciferol) and VD_3_ [6]. VD undergoes hydroxylation by hepatic 25‐hydroxylase (*CYP2R1*) to generate 25‐hydroxyvitamin D [25(OH)D], the main biomarker of VD status. Subsequently, 25(OH)D undergoes conversion to the biologically active hormone 1,25(OH)_2_D_3_ by renal 1*α*‐hydroxylase (*CYP27B1*). Furthermore, *CYP27B1* expression has been identified in extrarenal tissues, such as skin, intestinal epithelial cells, parathyroid glands, prostate, and breast tissues, facilitating local 1,25(OH)_2_D_3_ synthesis with autocrine or paracrine functionalities. VD metabolites predominantly circulate in plasma bound to albumin and VD‐binding protein (DBP). Ultimately, these metabolites are catabolized by 24‐hydroxylase (*CYP24A1*) and eliminated via urine [7, 8]. Furthermore, VD metabolism is influenced by genetic polymorphisms affecting enzymes such as 7‐DHC reductase, DBP, and hydroxylases, thus contributing to variability in circulating VD concentrations.

Serum concentrations of 25(OH)D below the threshold of 50 nmol/L (20 ng/mL) are generally considered indicative of VD deficiency, which directly contributes to bone disorders like osteoporosis and rickets. Furthermore, low VD levels exhibit inverse correlations with the occurrence of chronic disorders, including type 2 diabetes and specific malignancies [9]. Additionally, VD deficiency has been associated with heightened susceptibility to various autoimmune disorders, such as Sjögren’s syndrome (SS), systemic sclerosis, and Behçet’s disease [10–12]. Given the global variability in VD status and the feasibility of supplementation, some experts have proposed VD supplementation as a viable public health intervention [13]. Currently, daily oral supplementation of ~800–1000 IU of cholecalciferol is recommended for healthy individuals [14]. Global epidemiological studies indicate a higher prevalence (ranging from 24% to 49%) of severe VD deficiency (serum 25(OH)D < 25 nmol/L) in Europe, Asia, and Africa compared with Latin America, Oceania, and North America [15]. A meta‐analysis reported a 20.93% prevalence rate of severe VD deficiency in Asian populations [16].

**Figure 1:**
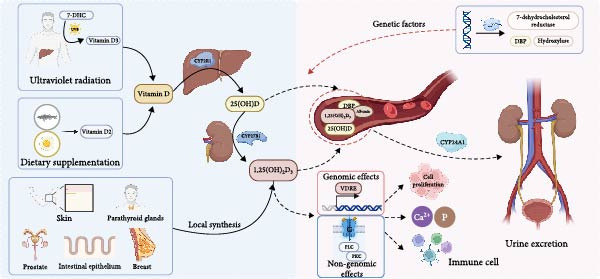
Metabolic pathway of vitamin D.

VDR, a nuclear hormone receptor extensively expressed in diverse tissues, mediates many biological effects of VD, particularly in immune regulation [17]. The biological effects mediated by 1,25(OH)_2_D_3_ via VDR involve both genomic and nongenomic pathways. Genomic signaling entails the direct binding of VDR to VD response elements (VDREs), influencing transcriptional activity of ~3% of the human genome. Conversely, rapid nongenomic effects occur through membrane‐associated proteins, notably 1,25D‐MARRSBP (ERp57), initiating intracellular signaling cascades like the G protein‐phospholipase C (PLC)‐protein kinase C (PKC) pathway, which governs calcium flux and cellular proliferation. Hence, the actions of 1,25(OH)_2_D_3_ through both genomic and nongenomic mechanisms underscore its multifaceted role in modulating calcium‐phosphate balance, immune responses, and cellular differentiation and proliferation [18]. This mechanistic complexity highlights VD’s therapeutic potential in disease intervention and preventive healthcare strategies. (Figure 1).

## 3. Association Between VD and ITP

Numerous clinical studies have demonstrated that VD levels are generally reduced in both adult and pediatric patients with ITP. Cekerevac et al. retrospectively analyzed 152 children with chronic ITP in the Republic of Serbia, reporting that only 3 patients exhibited sufficient serum VD levels. Approximately 25% of patients had VD levels ranging from 20 to 30 ng/mL, whereas the remainder presented levels below 20 ng/mL, with 17% below 10 ng/mL [19]. Fattizzo et al. examined 103 patients with autoimmune cytopenias, including primary autoimmune hemolytic anemia (AIHA), Evans syndrome, chronic neutropenia, and ITP, comparing them with 40 age‐ and sex‐matched healthy controls. The results demonstrated that among the enrolled patients, the proportion of those diagnosed with ITP was the highest, reaching 42.7%. Moreover, the mean levels of VD detected in patients diagnosed with ITP, AIHA, and chronic neutropenia were lower relative to the healthy control group [20].

Hemorrhagic symptoms frequently occur as prominent clinical signs in ITP patients. Several studies have examined the potential relationship between VD status and bleeding manifestations. For instance, a clinical investigation involving 45 pediatric patients newly diagnosed with ITP from coastal Croatia assessed hemorrhagic severity using both the Skin–Mucosa–Organ Grading (SMOG) scale and the ITP Bleeding Scale (IBLS). This study found that lower serum 25(OH)D levels were associated with higher bleeding scores, indicating increased severity in skin and organ bleeding episodes [21]. However, geographical factors should be considered, as coastal regions receive greater sunlight exposure, potentially resulting in relatively higher VD levels compared to other areas. Besides bleeding, fatigue is another distressing symptom that persists through the onset, treatment, and recovery phases of ITP. A cross‐sectional study investigating factors associated with fatigue in ITP patients revealed that those with VD deficiency experienced significantly greater fatigue severity. Using several multivariable models with clustered factors, it was concluded that VD alone accounted for 12% of the variance in fatigue severity [22]. Additionally, studies have documented seasonal fluctuations in VD levels. A retrospective cohort study analyzing VD status in adult and pediatric ITP patients found significantly higher rates of deficiency during winter and spring compared to summer and autumn [23].

Regarding the prognostic value of VD levels in ITP, Čulić et al. [24] evaluated serum VD concentrations among 21 Croatian pediatric patients, either newly diagnosed or with chronic disease. They observed a notably high prevalence of VD deficiency (85.7%), with significantly higher VD levels in newly diagnosed cases compared to patients suffering from chronic ITP. Conversely, differing outcomes were presented by Lassandro et al., who monitored VD concentrations among 30 pediatric ITP patients (16 chronic cases and 14 newly diagnosed). They found no significant difference in median VD levels between these two patient groups. These results suggest that VD deficiency may not necessarily be a chronic condition nor correlated with ITP disease progression [25]. Given the small sample sizes and absence of healthy controls in these studies, their conclusions must be interpreted cautiously.

Concerning VD’s prognostic significance in relation to ITP, Mabrouk et al. conducted a case‐control analysis involving pediatric ITP patients, investigating serum VD concentrations and their correlation with disease severity and therapeutic responsiveness. This investigation enrolled 50 children with chronic or persistent ITP and an equal number of healthy matched controls, using platelet counts and the SMOG bleeding scale to gauge severity. The findings revealed significantly reduced median serum VD concentrations in pediatric ITP patients compared with the healthy group. Notably, complete disease remission was achieved in 44% of children, all belonging to the VD‐sufficient group. Furthermore, VD‐sufficient patients exhibited significantly higher platelet counts, suggesting an association between adequate VD levels and improved treatment response and reduced disease severity [26]. Similarly, Liang et al. analyzed clinical data from 160 pediatric ITP patients to identify prognostic factors. Through univariate and multivariate regression analyses, initial platelet count, predisposing factors, and levels of 25(OH)D_3_, C3, and IgG were identified as correlates of remission rates in pediatric ITP patients [27]. In summary, despite some discrepancies among studies, the findings consistently indicate that VD deficiency is prevalent among both pediatric and adult patients with ITP. Moreover, VD deficiency appears to correlate with clinical symptoms, treatment response, and prognosis in ITP patients.

VDR, a nuclear receptor that mediates the biological effects of 1,25(OH)_2_D_3_, has also attracted considerable interest. Mu et al. [28] identified significantly decreased peripheral blood levels of both 25(OH)D_3_ and 1,25(OH)_2_D_3_ in ITP patients, with a concurrent notable increase in VDR mRNA expression levels. Liu et al. [29] further highlighted significant inverse correlations between VDR mRNA expression and serum 25(OH)D_3_ and 1,25(OH)_2_D_3_. Additionally, CYP27B1, the gene encoding the key enzyme responsible for VD activation, plays a fundamental role in VD metabolism [29]. Another relevant study indicated significantly reduced expression levels of VDR and CYP27B1 mRNA in active ITP patients, with a positive correlation between these mRNA levels and platelet counts [30].

## 4. Potential Mechanisms of VD in ITP

The complex pathogenesis underlying ITP is closely linked to dysregulated humoral and cellular immune responses. VD, recognized as an essential immune regulator, substantially influences the immunopathological pathways in ITP by modulating the differentiation, maturation, and function of dendritic cells (DCs), B cells, and T cells, and maintaining cytokine network homeostasis. Furthermore, accumulating evidence suggests a notable association between specific polymorphisms in the VDR gene and increased risk and progression of ITP.

### 4.1. B Cells

Autoantibodies produced by B cells can not only accelerate platelet destruction but also impair megakaryocyte maturation, consequently reducing platelet production. Abnormal humoral immune responses mediated by B cells remain widely recognized as the primary pathogenic mechanism in ITP [31]. The biologically active metabolite of VD, 1,25(OH)_2_D_3_, can suppress activated B‐cell proliferation, inhibit their differentiation into antibody‐secreting plasma cells, and decrease IgG and IgM production, ultimately reducing autoantibody synthesis and attenuating platelet destruction. Moreover, 1,25(OH)_2_D_3_ can facilitate apoptosis and diminish survival of activated B lymphocytes [32, 33]. Li et al. [34] reported markedly reduced serum 25(OH)D_3_ concentrations in ITP patients. Pearson correlation analysis revealed significant negative associations between serum 25(OH)D_3_ and platelet surface markers, including platelet‐associated immunoglobulin M (PAIgM), platelet‐associated IgG (PAIgG), and platelet‐associated complement C3 (PAC3). Conversely, peripheral blood lymphocyte VDR levels were positively correlated with these platelet‐associated markers (PAIgM, PAIgG, and PAC3) [34].

B‐cell activating factor (BAFF) and its homologous molecule APRIL (a proliferation‐inducing ligand) exist as trimeric proteins within the tumor necrosis factor (TNF) superfamily. In ITP pathogenesis, the interaction between BAFF and its receptor (BAFF‐R) not only diminishes apoptosis rates in B cells and CD8+ T lymphocytes via induction of anti‐apoptotic proteins BCL‐2 and BCL‐xl, but also facilitates B‐cell proliferation, accelerates regulatory T cell (Treg) apoptosis, and enhances differentiation and expansion of Th17 cells [35, 36]. Studies have shown that serum APRIL levels correlate with disease activity in ITP patients. Data from a Chinese study comparing plasma APRIL concentrations between ITP patients and 30 healthy controls demonstrated significantly elevated APRIL levels in ITP patients, with active‐phase patients exhibiting a significant inverse correlation between APRIL concentrations and platelet counts [37]. Kamhieh‐Milz et al. further observed higher APRIL concentrations in active ITP patients. The same study measured BAFF levels, revealing elevated serum BAFF in active ITP patients relative to healthy controls. While glucocorticoid therapy reduced BAFF levels without affecting serum APRIL concentrations, the combined administration of prednisolone and VD_3_ synergistically lowered APRIL levels. These findings suggest that abnormalities in the BAFF/APRIL pathway contribute to ITP pathogenesis and that different therapeutic interventions exert distinct regulatory effects on this pathway [38].

### 4.2. T Cells

T lymphocyte‐mediated immune disturbances critically contribute to the pathogenesis of ITP. Patients with ITP commonly display a disrupted Th1/Th2 cellular equilibrium, heightened Th17 cell populations, increased IL‐17 secretion, as well as decreased numbers and defective regulatory function of Tregs, collectively leading to compromised immunological tolerance. The disrupted balance between Th17 and Treg subsets further accelerates autoantibody production through cytokines such as IL‐21, whereas hyperactivation of follicular helper T cells (Tfh) aggravates B‐cell differentiation and the subsequent generation of platelet‐specific autoantibodies [39, 40]. Additionally, CD8+ T cells contribute to platelet destruction not only by directly lysing platelets or inhibiting megakaryocyte function but also through certain subsets, such as regulatory CD8+ T cells (CD8+ Tregs), which may exert protective effects by inhibiting phagocyte activation and suppressing autoreactive T/B cell proliferation [41]. Furthermore, abnormal lipid raft structures and enhanced T cell receptor (TCR) signaling observed in T cells from ITP patients further amplify autoimmune responses [42]. VD directly regulates T cell function through VDR‐mediated signaling, significantly reducing IFN‐*γ* production and suppressing Th1 cell differentiation, while simultaneously promoting Th2 cell development and enhancing secretion of IL‐4, IL‐5, and IL‐10, thus modulating the Th1/Th2 balance [43, 44]. Moreover, VDR signaling attenuates Th1‐ and Th17‐mediated autoimmune responses by inhibiting proinflammatory cytokines, such as IFN‐*γ* and IL‐17, while potentially enhancing Th2 and Treg functionality indirectly to correct immune dysregulation [45].

Yao et al. investigated serum 25(OH)D_3_ levels alongside T cell‐related cytokine profiles among 127 pediatric patients diagnosed with ITP. Their findings showed significantly diminished 25(OH)D_3_ concentrations accompanied by elevated serum IL‐2 and IL‐17, in contrast to decreased levels of IL‐4 and IL‐10, when compared with healthy controls [46]. Similarly, Yang et al. [47] examined Th17/Treg ratios as well as IL‐17 and TGF‐*β* levels in 30 ITP patients and 30 matched healthy individuals, observing that ITP patients had elevated peripheral Th17 cell percentages, increased expression of HMGB1 and IL‐17, reduced proportions of Tregs, and decreased TGF‐*β* secretion relative to healthy subjects. Furthermore, Ouyang et al. investigated associations between serum 25(OH)D_3_ status and Th17/Treg cytokines in 32 pediatric Chinese ITP patients, categorized by VD status into deficiency (<20 ng/mL), insufficiency (20–30 ng/mL), and normal groups, alongside matched controls. Their results demonstrated significantly lower serum VD_3_, IL‐10, and TGF‐*β* levels in ITP patients compared to healthy controls, whereas IL‐17 and IL‐21 concentrations were markedly elevated. Further analysis of cytokine profiles and platelet counts across different VD status groups revealed that the deficiency group exhibited the most pronounced abnormalities, with significantly elevated IL‐17 and IL‐21 levels, lower IL‐10 and TGF‐*β* concentrations, and decreased platelet counts compared to other groups, demonstrating statistically significant intergroup differences [48].

### 4.3. Dendritic Cells

Dendritic Cells (DCs) play dual roles in autoimmune responses characteristic of ITP, acting not only as antigen‐presenting cells to activate T lymphocytes but also potentially serving regulatory purposes. In ITP patients, monocyte‐derived DCs (moDCs) exhibit enhanced phagocytic capacity toward apoptotic platelets, heightened activation of autoreactive T cells, and display a distinctly proinflammatory phenotype characterized by increased expression of costimulatory molecules (CD80/CD86) and augmented IL‐12 secretion, consequently driving Th1 differentiation [49, 50]. Simultaneously, the immunoregulatory properties of DCs become compromised in ITP. Normally, tolerogenic DCs maintain immune equilibrium through indoleamine 2,3‐dioxygenase (IDO)‐mediated tryptophan metabolism and subsequent induction of Tregs. However, moDCs from ITP patients exhibit reduced IDO expression, thereby impairing their capacity to induce naïve CD4+ T‐cell differentiation into functional Tregs, significantly diminishing their regulatory effectiveness [51]. DC populations include myeloid DCs (mDCs) and plasmacytoid DCs (pDCs); mDCs typically direct differentiation of naïve TH0 cells toward a TH1 lineage, whereas pDCs preferentially promote TH2 differentiation. ITP typically exhibits a Th1/Th2 imbalance with predominant Th1 responses. Li et al. [52] investigated peripheral DC subsets in ITP patients, observing increased mDCs and decreased pDCs in circulation. Another study comparing 26 treatment‐naïve adult chronic ITP patients with healthy controls found comparable numbers of mDCs and pDCs between groups, but significantly lower percentages of Tregs in patients. Following high‐dose dexamethasone (HD‐DXM) treatment for 4 days, marked increases in Tregs and mDCs and significant reductions in pDCs were observed [53].

### 4.4. VDR Genetic Polymorphisms

Located on chromosome 12q13.11, the VDR gene encodes a nuclear receptor protein that functions as the central mediator of VD signaling. The gene spans around 100 kilobases, encoding a receptor composed of 424–427 amino acids. Among the more than 470 identified single‐nucleotide polymorphisms (SNPs) within the VDR gene, four classical polymorphic sites, *FokI* (rs2228570 T/C), *BsmI* (rs1544410 A/G), *ApaI* (rs7975232 C/A), and *TaqI* (rs731236 T/C), have been extensively analyzed and reported in the literature [54]. These VDR polymorphisms modulate receptor functionality and influence the biological effects of VD, making them promising molecular markers for personalized nutritional interventions and disease risk assessment. A meta‐analysis demonstrated that *TaqI* and *FokI* polymorphisms may influence individual responses to VD supplementation by altering receptor function, with Tt/tt (*TaqI*) and FF (*FokI*) genotypes exhibiting more pronounced beneficial effects. In contrast, *BsmI* and *ApaI* polymorphisms showed no significant impact, providing a potential genetic basis for individualized VD supplementation [55].

In recent years, increasing attention has been directed toward elucidating the association between VDR gene polymorphisms and susceptibility to ITP. A study conducted on a Turkish cohort assessed the functional promoter polymorphism *CDX2* (rs11568820), along with the previously mentioned polymorphisms (*FokI*, *BsmI*, *ApaI*, and *TaqI*), exploring their potential involvement in the development of ITP. The study demonstrated for the first time a significant correlation between the *CDX2* polymorphism and pediatric ITP risk, showing that the GG genotype increased susceptibility, while the A allele conferred protective effects. However, no significant associations were identified for *FokI*, *BsmI*, *ApaI*, or *TaqI* polymorphisms [56]. Shaheen et al. further reported that, among Egyptian pediatric and adolescent ITP patients, the CC genotype of VDR *FokI* polymorphism was associated with higher serum VD levels, whereas the *BsmI* G allele (GG/AG genotypes) approximately doubled ITP risk. Nonetheless, no differences in VDR polymorphisms were observed between newly diagnosed and chronic ITP cases. The authors suggested that lifestyle changes accompanying socioeconomic development in Egypt might interact with VDR polymorphisms, highlighting gene–environment interactions as a potential mechanism in pediatric ITP pathogenesis [57]. In another investigation involving genetic analysis of 40 adult ITP patients and 60 ethnically and geographically matched healthy subjects, significant variations were observed in the distribution of *BsmI* genotypes. Notably, the bb genotype appeared to confer protective effects against the occurrence of ITP [58]. Additionally, Radwan et al. studied the correlation of VDR polymorphisms with steroid responsiveness in Egyptian ITP patients. Their findings indicated that the F allele of VDR *FokI* was significantly associated with decreased serum VD concentrations and resistance to corticosteroid treatment, whereas the haplotype *BAF/BAF* was exclusively identified in patients exhibiting steroid resistance. These results suggest that VDR polymorphisms, particularly *FokI*, may influence treatment responsiveness, providing genetic evidence supporting personalized therapeutic approaches [59]. Observed inconsistencies in VDR polymorphism distributions across studies may arise from differences in ethnic populations, sample sizes, environmental exposures, and geographical factors.

## 5. Therapeutic Applications of VD and Its Analogs in ITP Treatment

Due to their immunoregulatory roles implicated in ITP pathogenesis, VD and its analogs have gained attention as potential therapeutic options for ITP management. However, in contrast to other autoimmune or inflammatory conditions such as IBD, endometriosis, or systemic lupus erythematosus (SLE), studies evaluating the therapeutic efficacy of VD supplementation in ITP remain sparse, and evidence supporting its use is comparatively weaker.

Bockow reported two cases of refractory secondary ITP that were successfully treated with high‐dose VD supplementation combined with hydroxychloroquine. The first patient, a 79‐year‐old male with concurrent SLE and SS, presented with a platelet count as low as 9 × 10^9^/L that was unresponsive to high‐dose prednisone and IVIG. Following adjunctive treatment with hydroxychloroquine (400 mg/day) and high‐dose VD (50,000–150,000 IU/week), his platelet count rose to 141 × 10^9^/L. Notably, discontinuation of VD led to an abrupt decline to 18 × 10^9^/L, which rebounded upon resumption. The second patient, an 87‐year‐old female with overlapping SLE/SS and ITP, had a baseline platelet count of 8 × 10^9^/L and severe VD deficiency (17 ng/mL). After initiating hydroxychloroquine and VD (50,000–100,000 IU/week), her platelet count increased to 301 × 10^9^/L, maintaining sustained remission during long‐term follow‐up. Both cases demonstrated significant platelet recovery and reduced bleeding risks without developing hypercalcemia or renal dysfunction despite high‐dose VD treatment [60]. It is important to emphasize that the aforementioned cases represent secondary ITP associated with SLE and SS. While these findings highlight the therapeutic potential of VD in complex autoimmune contexts, their extrapolation to primary ITP requires caution. The immunopathological background of secondary ITP is driven by another systemic autoimmune disease, resulting in a broader scope of immune dysregulation that may differ significantly from isolated primary ITP. Therefore, future studies evaluating the efficacy of VD should clearly distinguish between these two distinct entities.

Liu et al. demonstrated that 1,25(OH)_2_D_3_ significantly suppressed the proliferation of peripheral blood mononuclear cells (PBMCs) derived from both healthy subjects and ITP patients, though no statistically significant difference in inhibitory effects was noted between the two groups. Their study showed that 1,25(OH)_2_D_3_ markedly reduced the Th1/Th2 and Tc1/Tc2 ratios in ITP patients, while increasing Treg proportions without significantly altering Th17 percentages. Furthermore, 1,25(OH)_2_D_3_ suppressed IFN‐*γ* and IL‐17A secretion in PBMC culture supernatants, while enhancing IL‐10 production, but exerted no significant effect on IgG, TNF‐*α*, or TGF‐*β*1 levels. To elucidate the effects of 1,25(OH)_2_D_3_ on T‐cell differentiation, the authors assessed transcription factor expression, observing upregulated GATA3 and Foxp3 mRNA levels alongside downregulated T‐bet mRNA in ITP patient‐derived PBMCs following 1,25(OH)_2_D_3_ treatment. These findings suggest that dysregulated 1,25(OH)_2_D_3_/VDR signaling may contribute to ITP pathogenesis [30].

Chinese researchers investigated whether 1,25(OH)_2_D_3_ could ameliorate ITP symptoms via immunomodulation in a mouse model. The study involved 44 BALB/c mice divided into normal control, ITP model, and treatment groups, with the latter group receiving daily tail‐vein injections of 100 ng 1,25(OH)_2_D_3_. Mice in the ITP model group displayed persistently low platelet counts with compensatory megakaryocyte proliferation but decreased platelet‐producing megakaryocytes in bone marrow. Starting from day 10, the treatment group showed significant recovery in platelet counts, lower bleeding scores, and reduced subcutaneous hemorrhage compared to model mice. Moreover, treated mice exhibited higher platelet‐producing megakaryocyte numbers relative to the model group (though still below normal), and fewer total megakaryocytes compared to model mice (but higher than controls). These observations indicate that 1,25(OH)_2_D_3_ may promote platelet production and alleviate bleeding symptoms [61]. This animal study underscores the therapeutic potential of 1,25(OH)_2_D_3_ for ITP, although clinical trials remain necessary to verify these results.

Li et al. 62] conducted several randomized controlled trials (RCTs) examining VD as adjunctive therapy in adults with ITP. In Study 1, 80 adult ITP patients were divided into an observation group receiving daily alfacalcidol plus prednisone and a control group treated with prednisone alone. After 6 weeks, both groups showed increased platelet counts and 1,25(OH)_2_D_3_ levels, alongside decreased lymphocyte VDR expression and reduced Th17 cell proportions; however, the improvements were more pronounced in the observation group [62]. In Study 2, 146 chronic ITP patients were randomized equally into observation and control groups; both received sirolimus capsules, with the observation group additionally receiving calcitriol capsules. After 6 weeks, the observation group exhibited greater elevation in 1,25(OH)_2_D_3_ levels, higher Treg cell counts, and improved FACIT‐F and ITP‐PAQ scores compared to controls [63]. Another study evaluated short‐ and long‐term outcomes in 90 newly diagnosed ITP patients (45 per group), comparing prednisone monotherapy with prednisone combined with VD_3_ drops. The combination therapy group achieved significantly faster complete response or improvement (6.48 ± 3.75 vs., 8.56 ± 4.61 days, *p*  < 0.05) and maintained superior sustained response rates with fewer relapses during a 6‐month follow‐up period [64].

## 6. Conclusions

This study systematically reviews the current state of research regarding VD in ITP, highlighting a close association between VD deficiency and the pathogenesis, disease activity, and treatment response in patients with ITP. VD deficiency is prevalent in ITP patients, and serum VD levels positively correlated with the severity of bleeding and fatigue symptoms, as well as treatment responsiveness. Mechanistically, VD regulates immune cell functions, including those of B cells, T cells, and DCs, through interactions between its active form 1,25(OH)_2_D_3_ and the VDR. In addition, VDR gene polymorphisms appear to influence susceptibility to ITP and therapeutic outcomes, providing a genetic basis for personalized treatment approaches. Animal models and clinical reports confirm that VD supplementation as adjunctive therapy can elevate platelet counts, ameliorate immune dysfunction, and improve patient prognosis.

However, significant heterogeneity exists among studies due to variations in ethnic background, environmental exposures, and geographic location. Current research into VD interventions in ITP remains limited, with no established consensus regarding optimal dosing strategies or treatment duration. Therefore, increased scholarly attention to VD’s therapeutic potential in ITP is urgently needed. Future research should focus on: (1) conducting multicenter RCTs to establish evidence‐based supplementation protocols; (2) exploring synergistic interactions between VD and existing therapeutic modalities; (3) performing rigorous mechanistic studies, utilizing both in vitro models and in vivo animal systems, to clarify underlying molecular mechanisms; and (4) developing precision treatment strategies guided by VDR genotyping. In conclusion, as a naturally occurring compound with both immunomodulatory properties and favorable safety profiles, VD represents a promising therapeutic agent for ITP management.

## Ethics Statement

The authors have nothing to report.

## Disclosure

All authors contributed to the article and approved the submitted version.

## Conflicts of Interest

The authors declare no conflicts of interest.

## Author Contributions

**Yucao Ma**: conceptualization, investigation, visualization, writing – original draft, writing – review and editing. **Wenjing Yao**: investigation, writing – review and editing. **Haiyan Lang**: supervision, writing – review and editing. **Yuxin Cheng**: investigation, visualization. **Ruhua Ren**: investigation, visualization. **Yuecan Chen**, **Sitong Cheng, and Shuo Sun**: visualization. **Qing Guo**: visualization, writing – review and editing. **Shana Chen**: visualization, writing – review and editing.

## Funding

This work was supported by the National Natural Science Foundation of China (Grant 82474467) and the Pilot Project for Enhancing Clinical Research and Achievement Transformation Capacity at Dongzhimen Hospital, the Beijing University of Chinese Medicine (Grant DZMG‐XZYY‐23004).

## Data Availability

The data supporting this review are from previously reported studies, which have been cited.
